# Supplementary material for: Lipids of Platelet-Rich Fibrin Reduce the Inflammatory Response in Mesenchymal Cells and Macrophages
Source: Cells. 2023 Feb 16;12(4):634. doi: 10.3390/cells12040634 (PMC9954017; doi:10.3390/cells12040634)
Supplement: Supplementary file 1 [file cells-12-00634-s001.zip › cells-2052334-supplementary.pdf]

Supplementary Materials: Lipids of Platelet-Rich Fibrin Reduce the Inflammatory Response in Mesenchymal Cells and Macrophages

Zahra Kargarpour , Layla Panahipour, Michael Mildner, Richard J. Miron and Reinhard Gruber

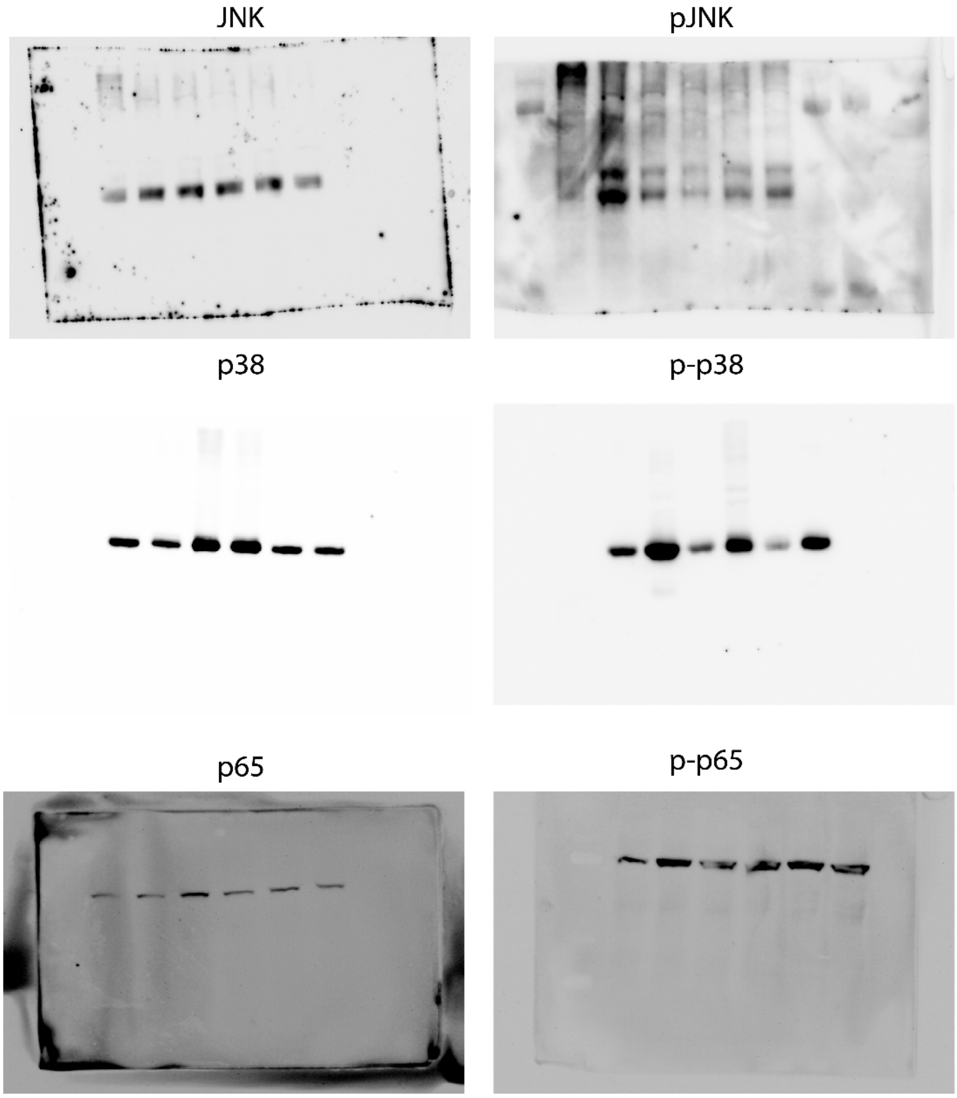

Figure S1. Uncropped WB images. Images show phosphorylated and non-phosphorylated antibodies JNK, p38 and p65. The grops from left to right are untreated control, TNFα+IL1β (TI), TI+LiPRF, TI+LiPPP, TI+LiBC, and TI+LiClot.
